# Recombinant PrP and Its Contribution to Research on Transmissible Spongiform Encephalopathies

**DOI:** 10.3390/pathogens6040067

**Published:** 2017-12-14

**Authors:** Jorge M. Charco, Hasier Eraña, Vanessa Venegas, Sandra García-Martínez, Rafael López-Moreno, Ezequiel González-Miranda, Miguel Ángel Pérez-Castro, Joaquín Castilla

**Affiliations:** 1 CIC bioGUNE, Parque Tecnológico de Bizkaia, 48160 Derio, Spain; jmoreno@cicbiogune.es (J.M.C.); herana@cicbiogune.es (H.E.); vvenegas@cicbiogune.es (V.V.); sgarcia@cicbiogune.es (S.G.-M.); rlopez@cicbiogune.es (R.L.-M.); egmiranda@cicbiogune.es (E.G.-M.); maperez@cicbiogune.es (M.Á.P.-C.); 2 IKERBASQUE, Basque Foundation for Science, 48011 Bilbao, Spain

**Keywords:** Prion disease, TSE, recombinant PrP, in vitro propagation, PMCA, QuIC

## Abstract

The misfolding of the cellular prion protein (PrP^C^) into the disease-associated isoform (PrP^Sc^) and its accumulation as amyloid fibrils in the central nervous system is one of the central events in transmissible spongiform encephalopathies (TSEs). Due to the proteinaceous nature of the causal agent the molecular mechanisms of misfolding, interspecies transmission, neurotoxicity and strain phenomenon remain mostly ill-defined or unknown. Significant advances were made using in vivo and in cellula models, but the limitations of these, primarily due to their inherent complexity and the small amounts of PrP^Sc^ that can be obtained, gave rise to the necessity of new model systems. The production of recombinant PrP using *E. coli* and subsequent induction of misfolding to the aberrant isoform using different techniques paved the way for the development of cell-free systems that complement the previous models. The generation of the first infectious recombinant prion proteins with identical properties of brain-derived PrP^Sc^ increased the value of cell-free systems for research on TSEs. The versatility and ease of implementation of these models have made them invaluable for the study of the molecular mechanisms of prion formation and propagation, and have enabled improvements in diagnosis, high-throughput screening of putative anti-prion compounds and the design of novel therapeutic strategies. Here, we provide an overview of the resultant advances in the prion field due to the development of recombinant PrP and its use in cell-free systems.

## 1. Introduction

Transmissible spongiform encephalopathies (TSEs) are a group of neurodegenerative disorders which have in common the formation of amyloid plaques due to the accumulation of prion protein (PrP) which has been converted to an abnormal conformation, known as PrP^Sc^, in the central nervous system (CNS). The misfolding of the normal cellular form of the PrP (PrP^C^) to the disease–associated form, PrP^Sc^, leads to neuronal damage, is invariably fatal but generally preceded by motor problems, such as myoclonus and ataxia, and by cognitive deficiencies. Different variants of TSE exist in many mammalian species [1]. In humans, five different prion diseases have been reported to date: Kuru [2], Gerstmann-Straüssler-Scheinker Syndrome (GSS) [3], Fatal Familial Insomnia (FFI) [4], Creutzfeldt-Jakob Disease (CJD) [5] and Variably Protease-sensitive Prionopathy (VPSPr) [6]. Each variant presents with distinct clinical signs and a different prion accumulation pattern in the brain. Besides human prion diseases, the best known examples due to the number of affected animals are: Scrapie in sheep and goat [7], Transmissible Mink Encephalopathy (TME) [8], Bovine Spongiform Encephalopathy (BSE) [9] and Chronic Wasting Disease (CWD) in cervids [10]. A slow virus was initially hypothesised to be the causal agent of these disorders. However, the lack of identification of any virus, despite extensive investigations and transmission of the disease after subjecting neural tissue to treatments known to inactivate nucleic acids, refuted this hypothesis and opened the possibility of another causal agent [11,12]. An alternative hypothesis was developed by S. Prusiner, known as the protein-only hypothesis. This controversial theory proposed that TSEs are caused solely by PrP^Sc^, a misfolded form of the physiologically normal PrP^C^ which is expressed abundantly in the CNS. This misfolded form of the protein is able to induce transformation of the normal PrP^C^ into a pathogenic conformation, initiating an infectious process in the brain of the affected individuals [13]. Due to the long incubation periods and phenotypic variability of prion disorders, reminiscent of virus strains, the existence of a pathogen devoid of nucleic acids was not widely accepted at first. However, the weight of evidence increased inexorably during the last three decades has proven irrefutably the proteinaceous nature of this infectious agent that defied the central dogma of the molecular biology [14]. According to the source of the PrP^Sc^ seed that initiate the infectious process, TSEs can be classified as: (1) acquired, when the PrP^Sc^ comes from an exogenous source [15,16,17]; (2) genetic, when the PrP^C^ misfolds due to mutations in the PrP encoding gene [18]; (3) or sporadic, when the cause is unknown although an spontaneous misfolding of the host’s wild type PrP^C^ is suspected [19]. The latter is the most common in humans, representing about 85% of the total cases [18]. 

The PrP is a glycosylphosphatidylinositol (GPI)-anchored membrane glycoprotein encoded by the *PRNP* gene, which is present in all superior animals and highly conserved in mammals. The native form of this protein is comprised of a mostly α-helical globular domain and a flexible amino terminal region [20,21]. The conformational change that results in transformation to the pathogenic isoform dramatically alters the biological and physicochemical properties of the PrP, which becomes neurotoxic, aggregation prone and partially resistant to protease digestion in most cases [22,23,24]. The details of this process remain largely unknown at the molecular level hindering the understanding of several aspects of TSEs. The main limitation comes from the impediment to unraveling the three-dimensional structure of the pathogenic conformer due to its amyloidogenic nature [25]. This hinders an adequate understanding of some of the most striking characteristics of prions such as the strain phenomenon, which is responsible of the existence of phenotypically distinct TSEs that share identical PrP sequences [26,27], or interspecies transmission of prions, since there is a transmission barrier between many species due to differences in their PrP amino acid sequences [7,28]. 

The study of TSEs and their causal agent has been limited for a long time to animal models naturally susceptible to prion diseases and started with Gajdusek and colleagues who demonstrated that both Kuru and CJD were infectious disorders by direct inoculations in the CNS of monkeys [29,30]. A similar approach was used to prove the relationship between BSE and variant CJD (vCJD) [31], and for the generation of rodent-adapted prions by inoculation of scrapie into mice [32]. The difficulties and costs associated with the maintenance, long incubation periods related to interspecies transmission barriers and the lack of ability to adapt and study certain prion strains significantly hindered progress in TSE research despite the advances achieved using naturally susceptible animal models. The emergence of the first transgenic mice expressing different PrPs [33] greatly increased the interest in animal models for research on prion diseases. These new models permitted evaluation of the transmissibility of different prion strains to transgenic animals bearing human PrP [34] and PrPs from other species [35] and also showed the effect of different *PRNP* gene mutations on the susceptibility to prion infection [36]. Moreover, models overexpressing PrP permitted shortening of the usually prolonged incubation times and facilitated obtaining large enough amounts of infectious material to study prions at the molecular level [35]. Nevertheless, generation of transgenic mice did not ameliorate all the problems related to animal models such as the high costs associated with their development and housing of high number of animals needed to reach valid conclusions. 

The development of cell cultures derived from different cell lineages all susceptible to prion infection addressed some of the limitations of the animal models and their use increased rapidly in the prion field [37]. However, most of the cell lines only propagate mouse-adapted prions in a highly strain-specific manner. In fact, different clones from the same cell line can show different susceptibility to the same prion strains [38] and cell lines highly susceptible to infection by some prions can be completely resistant to others [39]. Specificity issues were recently overcome by the development of non-neuronal cell lines [40] and these in vitro models are used to study several aspects of the cellular biology of prions including the native, non-pathogenic prion protein (PrP^C^). Nonetheless, developing cell models for prion infection is highly challenging and frequently unsuccessful [37].

Some of the problems associated with in vivo and in cellula models, primarily the limited quantity of PrP^Sc^ that could be obtained, were overcome in 1997 when Wüthrich and collaborators developed a novel technique to generate large amounts of recombinant PrP (rec-PrP) in *Escherichia coli* using a nickel-based purification system [41]. This technique enables the production of highly concentrated and pure rec-PrP for use in further investigations and has already proven its value in the study of TSEs. Despite the differences between brain-derived and recombinant PrP (the latter lacks glycosylations and GPI-anchoring to the cell membrane), it has enabled the atomic structure of the non-pathogenic PrP to be derived [21] and the development of several cell-free systems for prion generation in vitro [42,43,44,45,46,47,48,49,50,51]. Initially, in vitro generated misfolded protease-resistant PrPs (rec-PrP^res^) were poorly infectious in vivo [42], limiting their use as *bona fide* prion models. However, this situation changed with the generation of a highly infectious misfolded rec-PrP able to cause prion disease in vivo and reproduce all the characteristic hallmarks of a TSE [49]. Subsequently, recombinant PrP and cell-free systems for prion propagation have become invaluable tools. Herein, we focus on the different uses of rec-PrP and infectious misfolded rec-PrP and how their development has been pivotal in the field of TSE by enabling: mechanistic and structural studies, improvements in diagnosis and high-throughput screening of anti-prion compounds and the design of new therapeutic strategies (Table 1). 

## 2. Molecular Mechanisms

Compelling evidence has been gathered in support of the protein-only hypothesis of prion disease [13,14] resulting in the proteinaceous nature of the etiologic agent of TSEs accepted widely. Due to the novelty of a proteinaceous pathogen totally devoid of nucleic acid, the molecular mechanisms that lead to its transmission and propagation and the subsequent neurodegeneration were completely unknown. The first clues to the etiopathogeny of TSEs were from using animal models [29,52]. However, the complexity of these in vivo models limited the information that could be derived on the molecular mechanisms of the pathogen. Moreover, the long incubation times and the high costs associated with animal maintenance led to the search for a simpler, shorter term and more versatile models in order to make advances on the study of this unusual infectious agent. Cell culture models offered a simpler model that allowed unravelling several aspects of the cellular biology of the native PrP and the pathogenic isoform [53], although they are still complex models not totally suitable to study some aspects of the PrP such as tertiary structure or detailed misfolding mechanisms. The development of the first protocol to produce large amounts of pure, bacterially-expressed, recombinant PrP by Wüthrich and collaborators [41] provided a starting point for the development of cell-free systems and offered a new, reliable model that complemented the in vivo and in cellula models. The use of rec-PrP allows production of modified PrPs with chosen deletions, insertions, point mutations, distinct labeling and fusion proteins rapidly, providing versatility never seen before. Initially, the in vitro misfolded PrPs did not reproduce the hallmark characteristics of brain-derived PrP^Sc^, being either poorly infectious in vivo or totally non-infectious [42,43,44,45,46,47,48], limiting their usefulness in studying the native form of PrP. It is noteworthy that the first misfolded PrP produced in cell-free systems with minimal components was with brain-derived purified PrP^C^ and polyA RNA which resulted in a 50–100% substrate conversion efficiency [43] and demonstrated that fully infectious prions could be produced from purified components and cofactors. The production of the first misfolded rec-PrP infective in vivo (*bona fide* prion) by Ma and collaborators [49] demonstrated that it is possible to generate recombinant prions with all the hallmarks of PrP^Sc^ in cell-free systems, although the substrate conversion efficiency was <5% and the specific infectivity <100-fold lower than the previous recombinant prion. Finally, the first recombinant prion with high specific infectivity was produced a few years later using purified phosphatidylethanolamine (PE) as a cofactor instead of the lipids used by Ma and collaborators [54]. Therefore, at present, cell-free systems are the simplest model available to study several aspects of prion disorders that will be summarized in this section. 

Prior to obtaining infectious recombinant prions, rec-PrP and cell-free systems demonstrated their utility for (i) the search for molecules that could interact with the prion protein; (ii) studying the proneness to misfolding of different PrPs; and (iii) evaluating putative transmission barriers and the molecular mechanisms underlying these phenomena. The possible interactions of PrP with copper for example, have been a topic of interest as PrP has long been considered to play a central role in the homeostasis of copper in the CNS [55]. Its implication in prion propagation has also been reported [56,57], although its exact role in these process is unknown. The interaction of copper with octarepeat regions was known previously [58] but an additional domain was described using rec-PrP [59]. The effect of the interaction between copper and PrP and the misfolding process was also studied using rec-PrP-based cell-free systems [60,61,62]. The stability and misfolding proneness of distinct PrPs has been studied using rec-PrP and cell-free propagation systems too. Since the assessment of the influence of different domains and polymorphisms on the misfolding proneness of PrP usually requires modifying its primary structure, rec-PrP has been chosen as model for many of these studies due to the ease with which this can be accomplished. This is the case in research focused on unravelling the role of the N-terminal region of the PrP, which is intrinsically disordered [21], on misfolding and prion-associated neurotoxicity. Rec-PrPs with deletions in the N-terminal region have been used to show this domain is not necessary for PrP fibril formation, at least for mouse rec-PrP (amino acids 121-231) [63], hamster rec-PrP (90-231) [64], and human rec-PrP (90-231) [65]. Moreover, toxicity studies of the latter on cell cultures suggest that this domain is not directly involved in prion-associated neurotoxicity. The Central Lysine Cluster (CLC), encompassing amino acid residues 101-110 of the PrP, has been identified also as a critical region for the conversion to the pathogenic isoform due to the presence of several mutations associated with genetic prion disorders [66,67]. Specifically, recombinant hamster and mouse PrP were mutated to determine the effect of GSS-associated mutations in the CLC (P102L and P105L) on their proneness to misfolding. These mutations and others in the CLC were shown to play a pivotal role on the susceptibility of PrP to misfolding [68]. Similarly, the effect of different polymorphism that were suspected of influencing the susceptibility to prion infection in vivo have been confirmed in vitro using rec-PrPs. The susceptibility of humans to BSE infection is known to be influenced by a polymorphism at amino acid residue 129, where the presence of a methionine residue instead of a valine one results in increased susceptibility [69]. Experiments using human rec-PrP with both polymorphisms demonstrated that a methionine residue in position 129 makes the a α-helix more solvent-exposed, increasing its proneness to misfolding [70]. Polymorphisms in mouse PrP that define genotypes *a* (L108/T189) and *b* (F108/V189) were also known to influence significantly the pathogenesis upon infection with scrapie in vivo [71]. Recombinant prion fibrils obtained from both polymorphic forms of mouse rec-PrP determined a clear difference in the nucleation phase [72], clearly demonstrating the influence of these amino acid residues on the misfolding ability of PrP. Besides evaluating the effect of different protein regions and amino acid residues on the fibril formation process, the influence of chemical modifications can be tested easily also using rec-PrP. For instance, the role of methionine oxidation, an event that could be related to the ease of prion propagation [73,74] was proven using hamster rec-PrP, which upon methionine oxidation showed increased proneness for β-sheet structure acquisition [75] and fibril formation [76].

Despite most of the previous examples being performed using cell-free systems and giving rise to non- or poorly infectious amyloid fibrils composed of rec-PrP, their use for the investigation of possible molecular mechanism related to PrP misfolding is beyond doubt. However, the results from these methods need to be interpreted cautiously as they may not correlate completely with the molecular mechanisms that take place in vivo. For this reason, the generation of *bona fide* recombinant prions was a significant breakthrough in the field of TSE research. Nonetheless, even the methodology that allowed production of the first recombinant prions that were as infectious as those derived from brain tissue [49] show an intrinsic variability by producing different misfolded rec-PrPs with strikingly different biological properties [77]. This is most likely due to the generation of misfolded rec-PrP fibrils with distinct tertiary or quaternary structures in vitro, reminiscent of the different structures underlying different prion strains [78]. At present, this in vitro misfolding event cannot be controlled accurately thereby resulting in different misfolded rec-PrP conformations with different tertiary structures stochastically and depending on the conditions used [49,77,79]. Differences in the post-translational modifications between rec-PrP and brain-derived PrP probably hamper precise templating with brain-derived PrP^Sc^ seeds, impeding the formation of the same strains that can be found in vivo. Therefore, most of the misfolded rec-PrPs generated in vitro are non- or poorly infectious in animal models, despite showing other prion-like properties such as self-propagation or protease-resistance. In fact, prions obtained from mouse P101L recombinant PrP (the equivalent to P102L human mutation related to GSS) produced the accumulation of amyloid plaques in the CNS of TgP101L knock-in transgenic mice but no clinical signs were observed in these animals [80]. Therefore, it is important to consider the biological properties of the misfolded rec-PrPs in vivo in order to correctly interpret the significance of the results obtained with these models. Moreover, defining the factors that would allow controlling the biological properties of the misfolded rec-PrPs generated in cell-free systems is of utmost importance to obtain biologically significant and unrefutable conclusions regarding the molecular mechanisms under study. 

Legname and collaborators demonstrated that obtaining infectious recombinant prions is possible using purified rec-PrP, although challenge with this misfolded rec-PrP in a wild-type mouse model resulted in unusually long incubation periods [42]. This effect was probably due to the differences between the recombinant PrP and the mammalian cellular PrP, to the variety of conformations that the rec-PrP can acquire during its in vitro misfolding or to a lower infectious titer than brain-derived prions. In subsequent passages in animals, the misfolded rec-PrP adapted to in vivo propagation through a phenomenon that has been named deformed templating, which suggests that the initial slow propagation is due to incomplete structural compatibility [81]. Moreover, the generation of structurally distinct, misfolded rec-PrPs, which showed different biological properties, was demonstrated later. However, this set of ultrastructurally distinguishable recombinant prions converged into a common conformation upon successive inoculations in vivo [82]. The relevance of an appropriate three-dimensional structure over the differences between recombinant and mammalian PrP was proved by the generation of the first recombinant mouse prion capable of infecting wild-type mice with characteristics similar to those of brain-derived brain prions [49,83]. In this case, lipids (specifically POPG) and RNA were used as cofactors in the in vitro misfolding reaction, highlighting the relevance of appropriate cofactors to drive a mammalian prion-like misfolding, as addition of different cofactors rendered structurally highly similar but non-infectious prions under the same conditions [77]. This is in contrast with the observations of Deleault and collaborators that were able to obtain consistently recombinant prions with high specific infectivity using polyethylene glycol (PE) as a cofactor. The requirement of cofactor molecules for the formation of recombinant *bona fide* prions has been studied in depth and several molecules including specific lipids and polyanions were successfully used to obtain infectious recombinant prions [64,84,85,86,87,88]. However, the fact that absence of these cofactors restrict the rec-PrP misfolding towards recombinant *bona fide* prions has been undermined recently. Replication of the experiment that gave rise to the first highly infectious mouse recombinant prion [49] resulted in non-infective prions in vivo [79] suggesting that a stochastic element may be present. However, the generation of infectious recombinant prions was achieved recently in the absence of cofactors using both a murine full-length rec-PrP [47] and a C-terminally deleted rec-PrP (amino acids 23-144) [89]. These results demonstrated that obtaining recombinant prions with biological characteristics similar to mammalian prions is possible, although the conditions that invariably lead to this goal are not completely understood as yet. A clear example of this is the POPG-complemented recombinant prions [49] and PE-complemented ones [54] for which differences in specific infectivity and reproducibility could be due either to different protocols or to the different cofactors used. Despite not totally controlling the process, the ability to generate highly infectious recombinant prions, besides supporting the protein-only hypothesis, provides an invaluable model to finally unravel the greatest enigma of prions, their three-dimensional structure at a molecular level. 

Recombinant PrP and in vitro misfolded recombinant fibrils play a central role in several structural studies focused mainly on deciphering the changes occurring during the misfolding event. Although it is well known that changes in secondary and tertiary structure of the PrP occur during the misfolding event, the regions involved in the initial steps and the structure of the misfolded pathogenic protein are completely unknown, which is reflected clearly in the notably different structural models proposed [64,90]. Using bovine rec-PrP (amino acids 121-230) and high-resolution NMR the regional stability and structural changes occurring upon urea-induced misfolding were measured, revealing region-specific information about the initial steps of PrP misfolding [91]. Similarly, the fibrilization of mouse rec-PrP aggregated in vitro in the presence of chaotropic agents was followed by hydrogen-deuterium exchange and mass spectrometry, highlighting the significance of the C-terminal domain and the direct addition of rec-PrP monomers to the fibrils, without intermediate oligomeric states [92]. However, these studies were performed by inducing rec-PrP fibrilization with chaotropic agents, which may not reproduce the misfolding process that takes place in vivo. Cell-free systems that allow the generation of infectious *bona fide* prions contribute to solving this issue. In fact, self-propagating recombinant misfolded PrPs with distinct in vivo infectivity have been analyzed using distinct physicochemical techniques and revealed subtle structural differences in the regions 91-115 and 144-163 that could be responsible for the different infectivity [88]. Infectious amyloid fibrils composed of sheep rec-PrP were the first studied with high resolution techniques such as Atomic Force Microscopy (AFM) and solid state-NMR (ssNMR) and showed results were not in complete agreement with either of the structural models proposed for prions [93]. Apart from full-length rec-PrP, amyloid fibrils formed by C-terminal truncated recombinant prions from human, mouse and hamster were also analyzed by ssNMR, showing a parallel in-register β-core [89]. Collectively, these studies clearly show the potential of recombinant *bona fide* prions to finally obtain a high-resolution three-dimensional structure of prions.

## 3. Diagnosis

The similarity between the clinical signs of TSEs and other neurodegenerative diseases makes the early diagnosis of some prion disorders extremely difficult. Motor and cognitive alterations are common features in all neurodegenerative diseases, hampering definitive diagnosis. The analysis of molecular biomarkers such as the 14-3-3 protein in the cerebrospinal fluid (CSF) of suspicious cases is often used to support the diagnosis in addition to clinical signs. However, altered levels of such biomarkers is non-specific as it is a common trait in many neurodegenerative diseases [94]. Although early detection of PrP^Sc^ would be the best biomarker due to its specificity, the accumulation of detectable amounts of PrP^Sc^ is mainly restricted to the CNS and is a late event in the course of many prion disorders limiting its use as an early diagnostic tool [95,96]. Nonetheless, in the case of oral or intraperitoneal infections compelling evidence shows that minute amounts of PrP^Sc^ are present in some body fluids of the affected individuals and in some prion disease to a major extent in the lymphoreticular system. More importantly, these traits of PrP^Sc^ appear prior to its accumulation on the CNS during the pre-symptomatic stage of the disease [97,98]. The diagnosis of prion disorders in an early stage is of utmost importance to help distinguish them from other dementias [99]. This is also critical in terms of public health, since the presence of PrP^Sc^ in certain body fluids from pre-symptomatic individuals might be enough to infect others [100,101]. Thus, PrP^Sc^ detection is pivotal for biosafety in blood transfusions and surgery [102,103]. However, the exceptionally small amounts of the causal agent in easily accessible body fluids, such as blood or urine, restrict its direct detection [100,104]. In the case of the CSF, despite the specific infectivity could be much higher it is also low in the asymptomatic stage of the disease, making difficult to detect it directly [105]. In order to solve this problem two different strategies which use either PrP^Sc^ concentration or its amplification before detection have been adopted.

There are two main techniques for PrP^Sc^ concentration and detection in body fluids. Both take advantage of steel beads or wires to concentrate prions present in the blood and CSF, which for unknown reasons bind the PrP^Sc^ present in the sample [106,107]. The first one, called Direct Detection Assay (DDA), is based on direct detection of these concentrated (using a solid-state binding matrix) prions by specific antibodies [108]. The second, Standard Steel Binding Assay (SSBA) couples prion concentration with a Scrapie Cell Assay (SCA) on which PrP^Sc^ detection is based on the infection of susceptible cell lines using the concentrated prions as seeds [109,110]. However, the DDA relies on the direct detection of PrP^Sc^ after concentration which may not be present within the lower detection limit of the method depending on the disease stage of the affected individual. In the case of SSBA, the cell assay would act as reporter solving the issue of sufficient PrP^Sc^, although it is restricted to those prions for which susceptible cell lines exist. 

The development of cell-free systems using rec-PrP is the cornerstone of the detection methods based on PrP^Sc^ amplification and would overcome some of the limitations of the previous techniques. Several systems have emerged for in vitro prion propagation or amplification, all of them employing the use of a substrate that contains an excess of natively folded PrP and a small amount of PrP^Sc^ which acts as seed and promotes the misfolding of the PrP from the substrate. Subsequently, this larger amount of misfolded PrP can be detected directly using specific antibodies or amyloid-binding dyes. The development of protein misfolding cyclic amplification (PMCA) was a great step forward on this direction. Initially, PrP^C^ from brain homogenates was used as a substrate to amplify minute amounts of PrP^Sc^ from prion-infected tissue samples using serial cycles of incubation and sonication [111]. However, the use of brain homogenates as source of PrP limited its application to the existing animal models. Substitution of the PrP derived from brain homogenates for rec-PrP overcame some of these limitations [112]. Furthermore, any sequence of rec-PrP can be designed and generated including chimeric or mutant PrPs that could improve the sensitivity of the technique due to increases in proneness to misfolding. Caughey and collaborators used used hamster rec-PrP as substrate with PMCA to amplify PrP^Sc^ seeds obtained from the CSF of scrapie-infected hamsters [113]. This system permitted the detection of as little as 50 ag of PrP^Sc^, allowing the diagnose of scrapie in hamsters using just 2 µL of CSF. However, despite the improvement by using rec-PrP, this technique was never implemented in clinical practice as it could not be used successfully with human samples and because the complexity of the incubation/sonication system and the expertise needed to interpret the results precluded its daily use in hospitals. 

Another technique based on rec-PrP amplification to detect minute amounts of PrP^Sc^ is the Amyloid Seeding Assay (ASA). Instead of using natively folded rec-PrP and incubation/sonication cycles, rec-PrP is kept in a semi-denatured state through the addition of chaotropic agents, which is thought to be an intermediate state on its misfolding pathway leading to aggregation [114]. Under mild denaturing conditions (presence of 0.46 M of guanidine hydrochloride), constant temperature (37 °C) and shaking, a nucleation process takes place creating misfolded states of the rec-PrP, which is the first step in the growth of amyloid fibers that occurs later. The growth of the amyloid fibers is monitored measuring the Thioflavin T (ThT) fluorescence over time, which increases in intensity upon binding to amyloid fibers. This simplifies the detection compared to direct detection by western blotting and specific antibodies [115]. The misfolding of hamster rec-PrP into amyloid fibers in vitro that occurs under these conditions is a spontaneous and slow process with a lag phase (time needed to detect some fluorescence increase) of nearly 12 h. The addition of preformed amyloid fibers or PrP^Sc^ as seeds greatly accelerate rec-PrP fibril formation, reducing the lag phase to just 2 h. The sensitivity of the technique has been evaluated using purified fibers from phosphotungstenate (PTA)-precipitation of scrapie-infected mouse brains, hamster brains and sporadic CJD infected human brains. In all cases addition of a seed lead to accelerated ThT signal increase and estimates suggest that as little as 0.03 fg of PrP^Sc^ can be detected by ASA [115]. This technique would be more suitable as a practical diagnostic system because it does not require sonication and the rec-PrP is easily produced. Furthermore, the sensitivity is appropriate and the system requires less specialist knowledge and technical proficiency compared to PMCA. However, it has not been tested with CSF samples and the necessity of purified fibers or brain homogenate as seeds impede its use as a tool for early diagnosis of prion infections. 

To date, the most successful technique and the most likely to be implemented in clinical practice is based also on the use of rec-PrP as a substrate for prion amplification. The Real Time Quaking Induced Conversion (RT-QuIC) allows the conversion of rec-PrP into the protease-resistant misfolded PrP isoform (PrP^res^) using shaking and controlled temperature and avoids the use of chaotropic agents [116,117]. The fibril growth is measured in real time using ThT, which is present throughout the fibril growth. It has been used with multiple strains, species and different PrP^Sc^ sources, including different classical strains of hamster, human, bovine, cervine, ovine and murine prions and some atypical strains also and all of them are detected efficiently by this technique [118,119,120,121,122,123,124]. Moreover, it has been further simplified because a single rec-PrP (the one from bank vole, *Myodes glareolus*) has been found to act as nearly universal substrate for the detection of prion strains coming from different species, including human samples [122]. The use of this rec-PrP as substrate for RT-QuIC allows the accurate diagnosis of humans affected by sporadic prion disorders with 100% sensitivity and 100% specificity from nasal brushing samples [99,125,126]. This sampling technique is a painless and relatively non-invasive way to obtain neuronal samples from the olfactory bulb and this, coupled to the extremely sensitive and specific RT-QuIC, may become the routine diagnostic tool for prion disease diagnosis in pre-symptomatic patients in the future.

## 4. Screening

The unknown molecular mechanisms underlying prion propagation and the resultant neurodegeneration are motivating researchers in the field to seek chemical compounds with anti-prion properties empiricaly, mostly through high-throughput screening of large chemical libraries [127]. Despite some compounds with putative anti-prion features have been identified based on experimental evidence [128,129] or because of their amyloid binding properties [130,131,132], none of them was effective in vivo [133,134,135]. Therefore, the search for new compounds that may become effective treatments is an area of great activity in the TSE field, as well as the development of rapid, inexpensive and reliable systems for high-throughput screening. 

Although the most reliable results in the search for compounds with anti-prion properties would be by in vivo models, these are unsuitable for high-throughput screening due to the economical and ethical issues associated with the large number of animals required. Instead, cell culture models have been used to detect compounds that could impair prion propagation [136]. However, these models require expertise, have higher material costs than cell-free systems and are not suitable for human prion diseases as only some animal strains can be propagated. Moreover, despite being closer to the natural scenario, cell culture still does not result in all the characteristic hallmarks of a TSE and are slower than other in vitro systems because of the time needed to process samples and measure the decrease in PrP^Sc^ levels. Thus, the most cost-effective methods for high-throughput screening of putative anti-prion compounds relies on the use of rec-PrP. Relatively large quantities of pure rec-PrP can be produced easily and the protein can be modified or labelled in any way imaginable to facilitate the measurement of the outcome. Different in vitro techniques based on the use of rec-PrP have demonstrated their potential for the screening of putative anti-prion drugs. These techniques can be divided in two groups depending on the mechanisms sought to inhibit the progression of the disease; compounds able to bind to native PrP or those inhibiting fibril formation. 

Most of the techniques in use at present seek compounds able to bind to natively folded PrP and impede its misfolding through stabilization of this conformation or blocking putative interaction sites with the disease-associated isoform. This strategy was proven valuable due to compounds such as the cyclic tetrapyrroles [137] which were identified due to their capacity to bind proteins and alter their conformational properties. Some of these compounds have been shown to interact with PrP specifically and even the binding site of some have been identified by Nuclear Magnetic Resonance (NMR) [138] which appeared to be directly related to their ability to inhibit the formation of the misfolded isoform in vitro [139]. Similarly, low-molecular-weight heparin, which binds to rec-PrP and increases its thermal stability, was shown to inhibit fibrillization of rec-PrPs from mouse and hamster by RT-QuIC [140]. Although none of these compounds reached clinical trials due to toxicity or poor blood brain barrier permeability, these examples clearly demonstrate the utility of screening for compounds with the ability to bind to rec-PrP. Several assays have been developed in order to perform high-throughput screening of chemical compounds able to bind to rec-PrP.

The fluorescence polarization-based competitive binding assay uses rec-PrP and phosphorothioate oligonucleotides (PS-ONs) which are fluorophores that bind strongly to the protein and show certain pattern of fluorescence polarization (FP) when bound. The addition of compounds that compete with the PS-ON for the binding site displaces it changing the FP of the sample. The binding site of PS-ON has already been reported to be important as when it attaches to PrP it prevents misfolding [141]. Thus, any compound that competes for this binding site could be a good candidate for treatment of prion disorders. This system can be easily adapted for high-throughput screening of compounds as 96-well plates can be used and measurement of the outcome is rapid. This technique, combining the use of hamster rec-PrP and the fluorophore Randomer-FL, has been shown to be useful in screening for anti-prion compounds [141]. A similar technique was also developed based on FP technology using rec-PrP labelled with a different fluorophore, IANBD [*N*-((2-(iodoacetoxy)ethyl)-*N*-methyl)amino-7-nitrobenz-2-oxa-1,3-diazole) ester]. In this case, the proteins need to be mutated to include a cysteine residue on its primary structure without significantly altering its secondary and tertiary structure. The use of rec-PrP allows the generation of the required mutant PrPs and the measurement of secondary and tertiary structure by standard biophysical techniques. This approach was successfully used by Collinge and collaborators using a human rec-PrP with a cysteine substitution at position 145 for the screening of a 1200-compound library [142]. They identified a compound, Chicago Sky Blue 6B, which bound strongly to rec-PrP with anti-prion activity which was demonstrated also in cell culture. 

Surface Plasmon Resonance (SPR) is another technique that has demonstrated its potential for the high-throughput screening of anti-prion compounds through binding to rec-PrP. This technique is based on the measurement of changes that occur in the molecular weight of a protein upon binding of different molecules [143]. This screening system has been implemented successfully by Doh-Ura and collaborators using mouse rec-PrP (amino acids 121-231) and different compounds with known anti-prion capacity [144]. Propranolol was identified as a new anti-prion drug with the results being confirmed in cell-culture. 

The other group of techniques used for high-throughput screening of compounds aims to detect those which inhibit the misfolding or fibrillization of rec-PrP in cell-free systems, regardless of the mechanism of action of the compound. The generation of amyloid fibers in vitro using rec-PrP as a substrate was a breakthrough in the prion field as it was the first cell-free system [42] able to generate a highly infectious recombinant PrP [49]. From the diverse methods developed for rec-PrP fibrillization, some can be adapted to the high-throughput screening of compounds due to their technical characteristics. This is the case for the semi-automated cell-free system [145] and RT-QuIC [117], both easily automated due to the simplicity of the equipment needed and the possibility of robotising the measurement of the fluorescence outcome. Both techniques rely on an increase in Thioflavin T (ThT) fluorescence to detect the presence of fibrils formed by rec-PrP in the sample, which occurs spontaneously in both systems but can be accelerated by the addition of seeds. Therefore, these systems are suitable for high-throughput screening because they are easy to automate, have reduced costs compared to animal or cell culture models and are faster than any other system. The semi-automated cell-free system has demonstrated its ability to screen anti-prion drugs using mouse rec-PrP and different compounds with known anti-prion effects such as curcumin, PAMAM dendrimers and TMPyP-Fe(III) [145]. Similarly, RT-QuIC has been used successfully using human rec-PrP and CJD-affected brain homogenate samples as a seed. Among others, acridine, dextran sodium sulphate and tannic acid were used to provide proof of principle, showing the suitability of this system for high-throughput screening of compounds that could inhibit prion propagation in vitro [146].

Halfway between methods measuring binding of compounds to rec-PrP and those looking for inhibition of fibrillization of rec-PrP, the Scanning for Intensely Fluorescent Targets (SIFT) is worthy of mention. In this case, mouse rec-PrP labelled with a green fluorophore is incubated in the presence of CJD-affected brain homogenate and antibodies specific for human PrP labelled with a red fluorophore. The technique is based on measuring disturbances in the interactions between rec-PrP and the PrP^Sc^ present in a CJD-affected brain homogenate after the addition of compounds that could interfere with this interaction. In the absence of anti-prion compounds, rec-PrP and the CJD fibrils form ternary complexes resulting in a mix of red and green fluorescence, while addition of compounds that inhibit this interaction shifts the fluorescence emission [147]. This method was successfully used for screening ten thousand compounds and detected eighty hits from which six were effective in cell culture models.

Together, this work demonstrate the suitability and versatility of various systems based on rec-PrP for high-throughput screening of anti-prion drugs.

## 5. Therapy

At present, there is no effective treatment for TSEs and they remain invariably fatal. The lack of knowledge of the atomic structure of PrP^Sc^ and the molecular mechanisms leading to protein misfolding and spongiform degeneration impede the rational design of therapeutic approaches targeting PrP^Sc^ or the cascade that results in neurodegeneration. Despite this, many research groups have focused their efforts on finding a therapy for these devastating disorders. Although none of the strategies or compounds found as yet are suitable for clinical practice, several strategies have been designed with differing levels of success. The search for chemical compounds with anti-prion properties [127], discussed above, is the most obvious strategy. Other strategies proposed include gene therapy [148], administration of rec-PrPs which impair the misfolding of endogenous PrP [149,150], and immunological therapy aimed at inducing an immune response against prions [151,152]. Recombinant PrP plays a pivotal role in the last two therapeutic strategies showing its use in this area of TSE research. 

The existence of transmission barriers is a well-known phenomenon in the prion field that has led to the development of therapies involving the use of rec-PrPs able to interfere with the propagation of PrP^Sc^ at the expense of endogenous PrP. Transmission barriers were first identified when interspecies prion transmission was performed experimentally. Due to differences in the PrP amino acid sequences of prion-donor and receptor species, the transmission is hindered, as shown by prolonged incubation times and reduced attack rates (percentage of individuals that succumb to the disease) [153]. Upon subsequent challenges, with resultant brain material from individuals initially challenged, in the same receptor species, incubation times are shortened and the attack rate increased as a result of adaptation [154]. In addition to differences in PrP primary structure, differences in the three-dimensional structure of prion strains (even those with the same PrP sequence) can also result in transmission barriers [78,155]. This phenomenon, first observed in vivo, has been reproduced in vitro in cell-free systems mimicking prion misfolding. PMCA is a good example of this as a well-established method to study the transmission barriers using brain homogenates as substrate and different seeds derived from infected brain homogenates [156,157]. Using rec-PrP as substrate, it was found that differences in PrP amino acid sequence was not the only criteria imposing a transmission barrier, but that differences between brain-derived prions and rec-PrP (probably due to the absence of glycosylation and a GPI anchor) also hindered the in vitro propagation of brain-derived PrP^Sc^ [158]. Taking advantage of this phenomenon, administration of rec-PrP has been proposed as a direct treatment delaying prion misfolding and thus, the progression of the disease. Using PMCA with brain homogenates from transgenic mice overexpressing human PrP as substrate and CJD-affected patient brain homogenate as seed, it was shown that the addition of human rec-PrP to the system clearly impeded the propagation of the CJD seed in a dose-dependent manner [149]. Because of a possible adaptation of the brain-derived prions to the rec-PrP substrate, dominant negative PrPs have been sought, PrPs with lower capacity to be misfolded due to certain mutations [159], which could be the best candidates for this therapeutic approach. Although the exact molecular mechanism of the interference of rec-PrP with PrP^Sc^ is unknown, this approach was also shown to be effective in vivo. In this case, mice infected with rodent-adapted scrapie prions and treated with hamster rec-PrP administered intracerebrally showed a significant increase in the survival times in a dose-dependent manor, illustrating clearly that rec-PrP could be used as an inhibitor in the treatment of TSEs [160,161]. 

Another therapeutic strategy involving the use of rec-PrP is the immunological therapy. As the PrP is a ubiquitous protein abundantly expressed in the CNS and PrP^Sc^ shares the same primary structure, no immunological response arises against prions, a phenomenon known as autotolerance [162,163]. The aim of the immunological therapy is to overcome this autotolerance and induce the immune system to generate antibodies which upon binding to PrP could impede its conversion to the pathogenic isoform. For that purpose, rec-PrP is used like a vaccine antigen to induce the production of auto-antibodies. The effectiveness of the immunological therapy has been shown in different models. Using dimeric mouse rec-PrP expressed in *E. coli*, auto-antibodies were generated in mice which demonstrated their efficacy by inhibiting the formation of PrP^Sc^ in cell-culture models persistently infected with prions [164]. Mouse rec-PrP immunization was also shown to provide protection in vivo, delaying the development of the disease when used as a prophylactic therapy in mice [165,166,167]. Furthermore, immunization with rec-PrPs from other species, such as bovine rec-PrP, delayed the disease onset in mice inoculated with rodent-adapted prions [152]. Apart from mouse models, the ability of rec-PrPs to boost the immune system has been shown in hamsters immunized with recombinant hamster PrP [151]. Altogether these works demonstrated that the use of rec-PrP to promote an immune response against prions may be an effective prophylactic approach to prevent the development of TSEs.

Since the generation of the first recombinant misfolded PrP [42], several in vitro models have been developed based on this technology. Due to these techniques, different aspects of the molecular mechanisms responsible for prion misfolding were unravelled, important improvements in TSEs diagnosis were made, different anti-prion compounds were discovered and new therapeutic strategies were developed. The development and production of recombinant PrP has demonstrated its enormous potential for further understanding of these devastating prion disorders. 

## Figures and Tables

**Table 1:** Summary of the most relevant advances accomplished with rec-PrP in each research area.

TSE Research Area	Breakthrough	Reference
Molecular mechanisms	Production of highly pure bacterially-expressed recombinant PrP	[168]
	Determination of the 3D structure of cellular PrP	[21]
	Generation of the first infectious recombinant prions	[42]
	Generation of the first recombinant prions infectious in wild type animals	[49]
	Generation of the first highly infectious recombinant prions	[54]
	Interaction of PrP with copper confirmed	[59]
	N-terminal of PrP not necessary for misfolding	[63,64,65]
	Confirmation of increased misfolding proneness due to disease-associated mutations	[50,68]
	Generation of the first human infectious recombinant prions	[50]
	Confirmation of different misfolding proneness in polymorphic PrPs	[69,70,71]
	Description of possible mechanisms of strain generation and adaptation	[51,82]
	Description of the role of cofactors in the determination of biological properties	[47,49,51,55,78]
	Generation of models for 3D structure of recombinant misfolded PrP	[64,89,90]
Diagnosis	Development of PMCA based on rec-PrP for diagnosis from CSF	[113]
	Development of RT-QUIC for diagnosis from different body fluids and tissue samples	[99,122,126,135]
Screening	Development high-throughput screening methods	[144,145,147]
Therapy	Demonstration of dominant-negative effect of exogenous rec-PrP on the propagation of prions	[149,150]
	Immunotherapy based on injection of rec-PrP	[151,152]
